# Weakly Supervised MRI-Based Classification of Alzheimer’s Disease Using Clinical Pseudo-Labels

**DOI:** 10.3390/neurosci7050094

**Published:** 2026-08-24

**Authors:** Rong Xiao, Tingwei Quan, Xinglong Wu, Guoping Xu, Shangbin Chen

**Affiliations:** 1 Britton Chance Center for Biomedical Photonics, Wuhan National Laboratory for Optoelectronics, Huazhong University of Science and Technology, Wuhan 430074, China; xiaor@hust.edu.cn (R.X.); quantingwei@hust.edu.cn (T.Q.); 2 Hubei Key Laboratory of Intelligent Robot, School of Computer Science and Engineering, Wuhan Institute of Technology, Wuhan 430074, China; xwu@wit.edu.cn (X.W.); xugp@wit.edu.cn (G.X.)

**Keywords:** Alzheimer’s disease, weak supervision, structural MRI, pseudo-labeling, deep learning, external validation

## Abstract

Alzheimer’s disease (AD) classification from structural magnetic resonance imaging (MRI) may benefit from weak supervision that uses clinically meaningful but imperfect supervisory signals. We evaluated a weakly supervised framework in which a multilayer perceptron (MLP) trained on age, sex, and Mini-Mental State Examination (MMSE) scores generated clinical pseudo-labels to initialize a patch-based fully convolutional network (FCN). For 260 Alzheimer’s Disease Neuroimaging Initiative (ADNI) training participants, subsequent refinement combined 80% of the preceding MRI-model probability with 20% of the participant’s ground-truth diagnostic label. This design preserves a dominant pseudo-label/self-training component while using partial diagnostic guidance to stabilize refinement. The FCN generated whole-brain probability maps, and selected voxel probabilities were classified by a second MLP. The framework was developed using ADNI (*n* = 417). Using ADNI validation data only, iteration 3 and a classification threshold of 0.5 were selected and then applied unchanged to the held-out ADNI test set and the external AIBL (*n* = 182), FHS (*n* = 102), and NACC (*n* = 265) cohorts. The selected model achieved F1 scores of 0.853 in ADNI, 0.707 in AIBL, 0.765 in FHS, and 0.807 in NACC. These results support the feasibility and cross-cohort transferability of clinical pseudo-label-based weak supervision for MRI classification. The framework is not intended to be label-free; rather, it provides a transparent strategy for integrating imperfect clinical pseudo-labels with partially weighted diagnostic guidance during training.

## 1. Introduction

Alzheimer’s disease (AD) is the most common cause of dementia and is characterized by progressive cognitive decline and neurodegeneration. Cerebrospinal fluid biomarkers and amyloid or tau positron emission tomography can improve biological characterization [1,2,3], but their cost, availability, and invasiveness limit routine use. Structural magnetic resonance imaging (MRI) is widely available and can reveal patterns such as medial temporal and parietal atrophy; however, these findings are not sufficiently specific to establish an imaging-only diagnosis [4,5]. Consequently, AD assessment still depends on the integration of clinical evaluation, cognitive testing, and imaging.

Deep learning has enabled automated extraction of disease-related features from neuroimaging data [6]. Most MRI classifiers are trained using direct subject-level diagnostic labels, whereas weakly supervised learning uses supervision that is imperfect, partial, inexact, or noisy [7]. A recent *Nature* review similarly defines weakly supervised learning as leveraging “imperfect, partial or noisy forms of supervision” [8]. Following this broad usage, we describe the present framework as weakly supervised because the MRI network is not trained solely on direct diagnostic targets: it is initialized using clinically generated pseudo-labels, and subsequent refinement targets remain composite, combining model-generated probabilities with a partially weighted diagnostic component. We use the term weak supervision to describe this heterogeneous supervisory structure, not to imply label-free training.

Convolutional neural networks and fully convolutional networks can learn hierarchical representations from three-dimensional MRI volumes [9,10]. Their clinical utility nevertheless depends on transparent model development, leakage-free evaluation, and validation across cohorts that differ in scanners, acquisition protocols, and population characteristics [11,12,13]. Single-cohort performance may overestimate generalizability, and high-dimensional models can exploit site-specific or demographic correlates rather than disease-related neuroanatomy.

Qiu et al. previously demonstrated that predictions derived separately from structural MRI, MMSE, and logical-memory testing could be combined to improve NC-versus-MCI classification [14]. In a subsequent study, the same group developed a patch-based FCN–MLP framework that generated subject-specific AD probability maps and performed AD-versus-NC classification using MRI alone, clinical variables alone, or their multimodal fusion [15]. The model was validated across ADNI, Australian Imaging, Biomarkers and Lifestyle Flagship Study of Ageing (AIBL), Framingham Heart Study (FHS), and National Alzheimer’s Coordinating Center (NACC). The present study adopts this established imaging backbone but investigates a distinct supervision strategy in which clinically generated pseudo-labels initialize the imaging model and diagnosis-guided refinement subsequently anchors self-training.

Clinical variables such as age, sex, and MMSE can provide informative but imperfect supervision. In our framework, the clinical MLP converts these variables into initial pseudo-labels, thereby transferring clinically relevant information into MRI-model training without using the clinical variables as inference-time inputs. During refinement, the preceding MRI-model probability remains the dominant component (80%), whereas the true diagnosis contributes a 20% anchor to the updated target. This design preserves the weakly supervised/self-training character of the learning signal while limiting drift from clinically meaningful diagnostic boundaries. Final subject-level predictions are derived from MRI-model outputs.

Related work has explored weakly supervised attention, incomplete clinical scores, multi-task learning, and domain generalization for dementia classification and prognosis [16,17,18,19,20,21,22,23]. Our study complements these approaches by combining clinical pseudo-label initialization, diagnosis-guided self-training, an established FCN–MLP imaging backbone, and external evaluation across three independent cohorts. The principal methodological contribution is therefore the supervision design and transparent accounting of label use rather than a new imaging architecture.

Accordingly, this study evaluates a weakly supervised, clinically initialized and diagnosis-guided AD-versus-normal-cognition (NC) classifier. A clinical MLP generates pseudo-labels from age, sex, and MMSE; a patch-based FCN generates voxel-wise disease-probability maps from structural MRI; and a second MLP performs subject-level classification. The objective is to assess whether this composite weak-supervision strategy can support accurate MRI classification and transfer across independent cohorts while maintaining an explicit, auditable account of the diagnostic information used during training.

The principal contributions of this study are:

(1) Clinical pseudo-label initialization: an MLP trained on age, sex, and MMSE generates imperfect but informative subject-level targets for MRI-model training.

(2) Diagnosis-guided weak-supervision refinement: updated targets combine the preceding MRI-model probability (80%) with a partially weighted true AD/NC label (20%) for the 260 ADNI training participants.

(3) Validation-locked multi-cohort evaluation: iteration 3 and a threshold of 0.5 are selected using ADNI validation data and then applied unchanged to the held-out ADNI test set and to AIBL, FHS, and NACC without external-cohort retraining.

## 2. Materials and Methods

**Figure 1:**
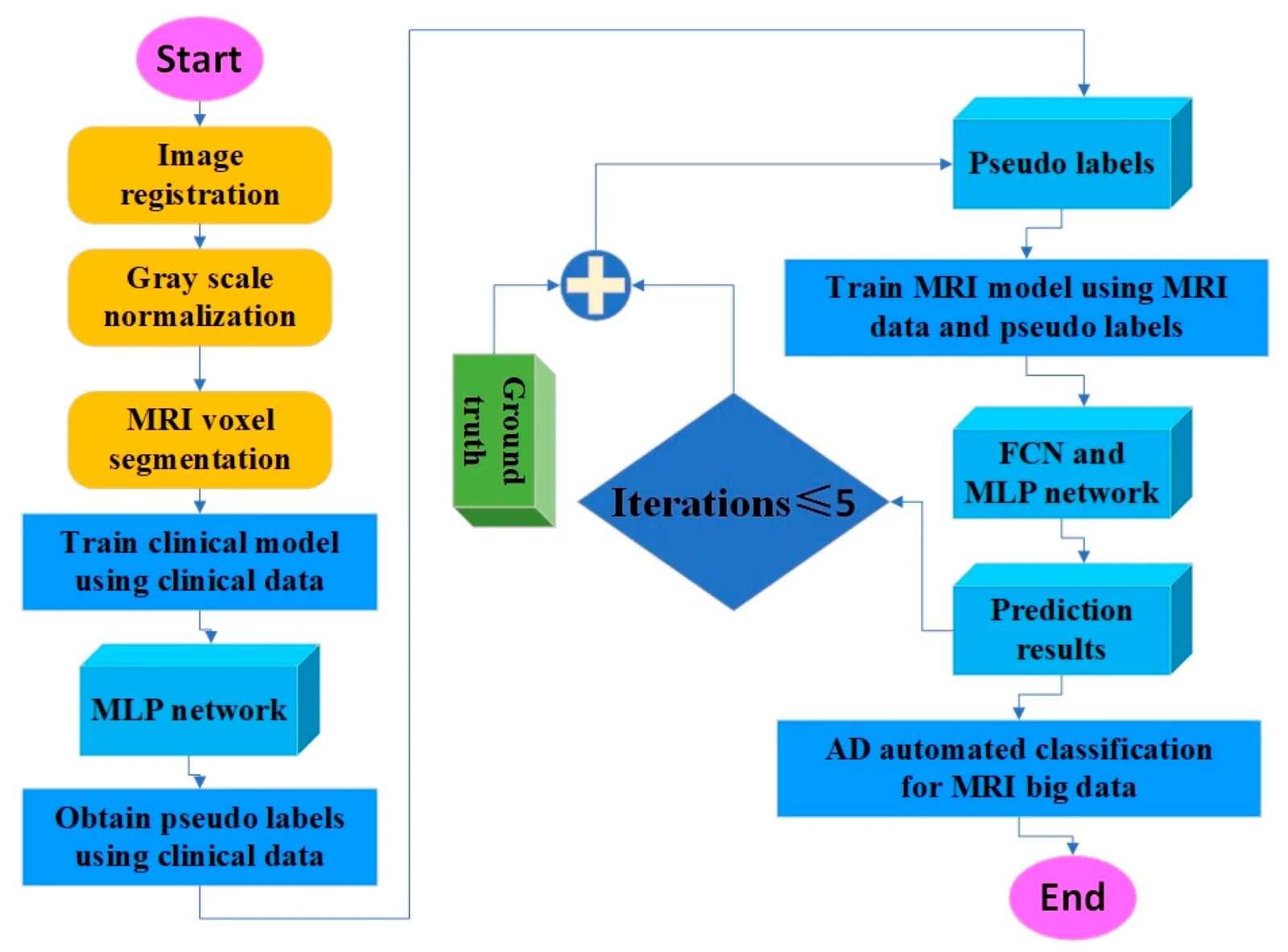
Overview of the weakly supervised framework for automated AD-versus-NC classification. Clinical variables generate initial pseudo-labels for MRI training, followed by diagnosis-guided refinement and locked evaluation on held-out and external cohorts.

The framework links a clinical MLP, a patch-based FCN, and an imaging MLP (Figure 1). The clinical MLP generates clinical pseudo-labels from age, sex, and MMSE. The FCN learns MRI patterns associated with these targets and produces a whole-brain disease-probability map. During later refinement rounds, the preceding MRI-model probability is combined with a partially weighted true AD/NC label from the ADNI training set. Selected voxel probabilities are then used by the imaging MLP for subject-level AD-versus-NC classification. Model development used ADNI (*n* = 417), whereas AIBL (*n* = 182), FHS (*n* = 102), and NACC (*n* = 265) were reserved for external evaluation.

### 2.1. Framework Overview

The proposed weakly supervised framework uses routinely collected clinical information to initialize MRI-model targets and then incorporates partially weighted ADNI training diagnoses during iterative refinement. All preprocessing, feature selection, checkpoint selection, and target refinement were restricted to ADNI development data; external cohort labels were reserved for final evaluation. Consistent with the broad definition of weak supervision as learning from imperfect or partial supervisory information [6], the framework combines clinically generated pseudo-labels, model-generated probabilities, and limited-weight diagnostic guidance while explicitly disclosing the role of each source.

The workflow consists of three stages: training a clinical model to generate pseudo-labels, training and refining the MRI model using composite weak-supervision targets, and applying the validation-selected MRI classifier to held-out and external cohorts.

First, the ADNI dataset (*n* = 417) was split at the participant level into training (*n* = 300), validation (*n* = 60), and testing (*n* = 57) subsets, corresponding approximately to a 5:1:1 allocation. From the training set, 40 participants (20 AD and 20 NC) were randomly selected to train the clinical MLP. The remaining 260 training participants received initial pseudo-labels generated from age, sex, and MMSE. Their diagnostic labels were not used to generate these initial pseudo-labels or to train the initial FCN, but were subsequently incorporated into each refinement target with a weight of 20%.

Second, MRI volumes from the 260 pseudo-labeled training participants were used to train the FCN, with each sampled patch inheriting its participant-level pseudo-label. At each refinement round, the resulting FCN probability was combined with the participant’s true diagnostic label to form the next composite target. The FCN was followed by an imaging MLP that converted selected disease-probability features into a subject-level AD/NC prediction.

To generate the target for the next refinement round, we used a hybrid weak-supervision update that combines self-training with partial diagnostic anchoring. Specifically, for the t-th refinement iteration, the updated pseudo-label probability for each subject in the ADNI training set was calculated as:

(1) $$P_{t}=0.8\times P_{FCN}^{\left(t-1\right)}+0.2\times Y_{GT}$$

where $P_{FCN}^{\left(t-1\right)}$ is the continuous AD probability output from the FCN in the previous iteration and $Y_{GT}$ is the true subject-level diagnostic label (0 for NC, 1 for AD). The fused probability was discretized at 0.5 to obtain the updated pseudo-label for the next refinement round, and the resulting hard target was used with cross-entropy loss. Thus, model-generated probability contributes 80% of the updated target and direct diagnostic supervision contributes 20%.

The 20% ground-truth component was applied only to the 260 pseudo-labeled participants in the ADNI training set. Diagnostic labels from the held-out ADNI test set and the external AIBL, FHS, and NACC cohorts were not used for training, feature selection, model selection, or threshold tuning; they were accessed only for final performance evaluation. This separation prevents test- or external-cohort labels from influencing model development.

In this study, “weakly supervised” is used in the broad sense of learning from imperfect or partial supervision [6]. The imaging model is initialized from clinical pseudo-labels and subsequently optimized using composite targets that retain an 80% model-generated component together with 20% diagnostic guidance. Accordingly, the framework is weakly supervised but not label-free. This terminology preserves the established meaning of weak supervision while making the exact data lineage and diagnostic-label contribution explicit.

**Figure 2:**
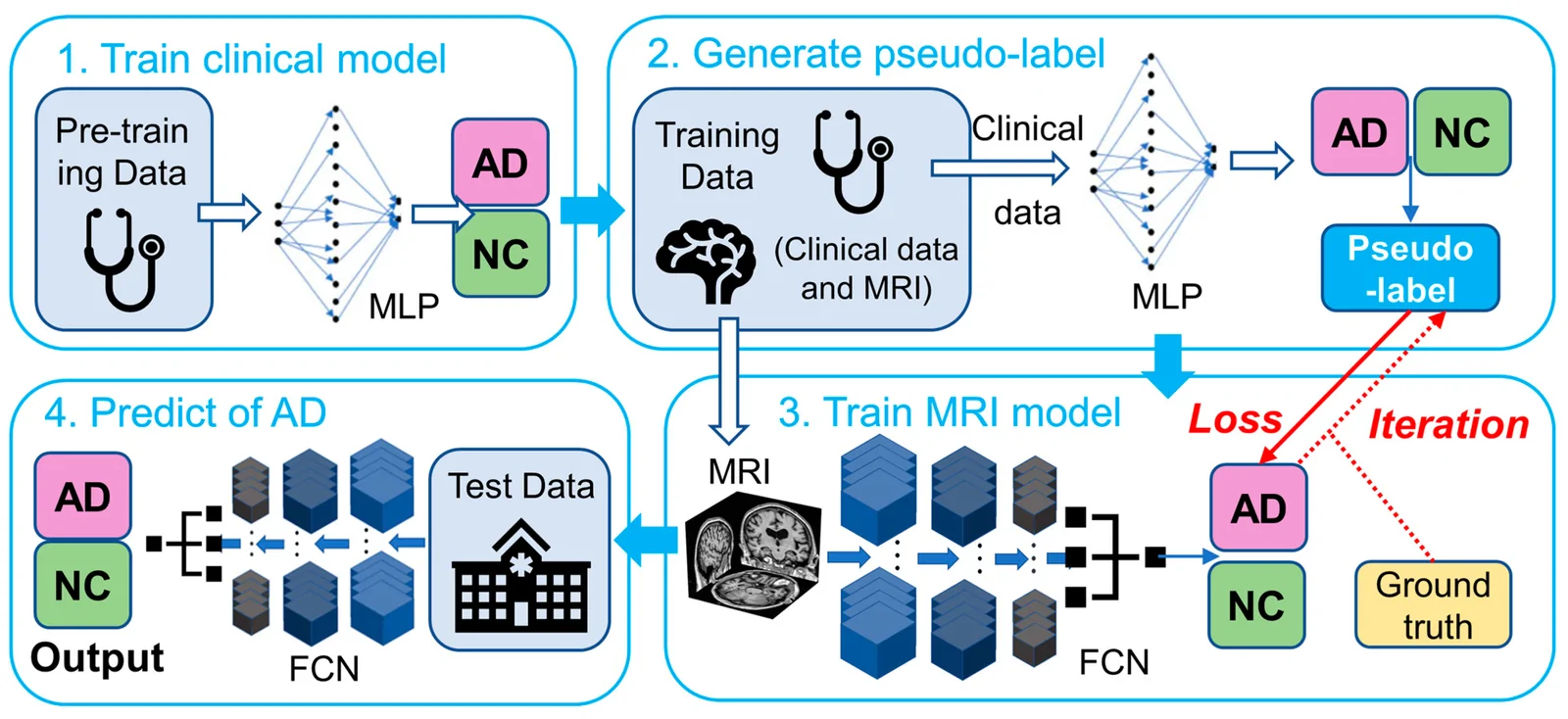
Architecture of the weakly supervised AD-classification framework. A clinical MLP generates initial pseudo-labels from age, sex, and MMSE. A patch-based FCN is initially trained on MRI volumes using these pseudo-labels and produces subject-specific disease-probability maps. During iterative refinement, the preceding MRI-model probability contributes 80% and the ground-truth AD/NC label contributes 20% to the updated target for the 260 ADNI training participants. Selected voxel probabilities are then passed to an imaging MLP for binary classification. AD, Alzheimer’s disease; NC, normal cognition; FCN, fully convolutional network; MLP, multilayer perceptron.

Five refinement iterations were evaluated. To avoid test-set-driven model selection, the primary model was chosen using ADNI validation data only: iteration 3 was selected because it achieved the highest validation F1 score, the checkpoint with the lowest validation loss was retained, and a classification threshold of 0.5 was selected on the validation set using the Youden index. This iteration, checkpoint, and threshold were then locked and applied unchanged to the held-out ADNI test set and all three external cohorts. Results from the remaining iterations are retained as sensitivity analyses and did not determine the primary conclusions, and Figure 2 illustrates the detailed architecture of the entire weakly supervised framework.

### 2.2. Data Lineage and Label Use

Table 1 summarizes the auditable data lineage for each model component and evaluation stage, specifying the participant subsets, input variables, diagnostic-label usage, and implementation purpose across clinical MLP training, initial pseudo-label generation, iterative refinement, validation, internal testing, and external evaluation. This transparent breakdown clarifies the exact contribution of clinical pseudo-labels, model-generated probabilities, and the partially weighted diagnostic anchor at each phase, ensuring full reproducibility of the supervision structure.

### 2.3. Network Architecture

We adapted the FCN architecture reported by Qiu et al. [15]. During FCN training, 3000 patches of size 47 × 47 × 47 voxels were randomly sampled from each participant’s full MRI volume without an anatomical-region restriction, providing broad whole-brain coverage. A weighted random sampler was used to balance classes within mini-batches. On-the-fly augmentation included random contrast, brightness, and additive Gaussian noise. Patches were shuffled during training; therefore, multiple patches from the same participant could contribute to a mini-batch and to its batch-normalization statistics.

Network weights were randomly initialized. The Adam optimizer was used with a learning rate of 1 × 10^−4^ and a batch size of 10. Each refinement round was trained for up to 100 epochs, with early stopping after 10 consecutive epochs without improvement in ADNI validation loss; the checkpoint with the lowest validation loss was retained. On an NVIDIA P5000 GPU, inference for one MRI volume required approximately 2 s.

All models were trained using cross-entropy loss with class weighting and no weight decay. Dropout was set to 0.5 for the main models, and PyTorch default weight initialization was used. Five training repeats were conducted using seeds 1000, 1001, 1002, 1003, and 1004. The archived analysis did not retain complete per-seed prediction files for every cohort; therefore, these repeats are reported as an implementation detail rather than used to construct formal run-to-run confidence intervals. The code was implemented in Python 3.6+ using PyTorch (≥1.1), NumPy (≥1.16), and standard scientific-computing libraries.

After disease-probability maps were generated, an imaging MLP performed binary classification using AD probabilities sampled from locations selected exclusively within the ADNI training partition. The MLP contained one hidden layer with a rectified linear unit activation, dropout, and a two-class output layer. A separate clinical MLP used age, sex, and MMSE as its three input features to generate the initial pseudo-labels. All imaging-model fitting and feature selection were confined to ADNI development data.

### 2.4. Data Preprocessing Pipeline

We preprocessed T1-weighted MRI data using skull stripping, affine registration, intensity normalization, and anatomical segmentation. Scans were stored in NIfTI format and aligned to the MNI152 template using FSL FLIRT with a 12-degree-of-freedom affine transformation and trilinear interpolation. Registrations were visually inspected; clearly misaligned scans (operationally defined as >10 mm shift or >5° rotation relative to the template) were manually corrected using anatomical landmarks. Such corrections were uncommon and occurred mainly in FHS (<10% of that cohort). Exact counts by cohort and diagnostic group were not retained in the original preprocessing log. Manual correction was performed by the first author without diagnostic blinding, and this is acknowledged as a limitation.

After registration and interpolation, intensity normalization was computed on the full scan volume before brain masking. Intensities were standardized to zero mean and unit variance, clipped to the range [−1, 2.5], and voxels outside the brain were set to −1 using the brain mask. FreeSurfer [24] segmentations were generated for a post-hoc neuropathological analysis in a small subset of FHS participants and were not used for FCN training, voxel selection, or the primary classification pipeline.

For subject-level classification, AD probability maps were generated for all 300 participants in the ADNI training partition using the trained FCN. A voxel-wise mean probability map was then computed within this training partition, and the 1000 locations with the highest mean AD probability were selected. Values at these fixed coordinates formed the input features for the imaging MLP.

Voxel selection was performed exclusively within the ADNI training partition before evaluation on the validation, internal-test, or external cohorts. The selected coordinates were frozen and applied identically to ADNI validation/test, AIBL, FHS, and NACC. This training-only feature-selection procedure prevents information from the held-out and external datasets from entering spatial feature selection.

## 3. Results

### 3.1. Datasets and Cohort Characteristics

This study included ADNI, AIBL, FHS, and NACC (Table 2). We intentionally used the cohort-defined clinical diagnostic groups supplied by each parent study, reflecting the diagnostic conventions under which these datasets were assembled. Because biomarker confirmation was not harmonized across cohorts, the task is best interpreted as classification of clinically defined AD and NC groups rather than direct detection of Alzheimer’s pathology. ADNI is a multicenter longitudinal study [25], AIBL investigates ageing and AD [26], FHS is a community-based longitudinal cohort [27], and NACC aggregates data from US Alzheimer’s Disease Research Centers [28].

Participants were included if they had at least one T1-weighted volumetric MRI scan acquired within 6 months of a recorded cohort diagnosis. Scans with fewer than 60 slices were excluded. When multiple eligible MRI–diagnosis pairs were available, the temporally closest pair was selected, and one scan per participant was analyzed. ADNI was split at the participant level into training (*n* = 300), validation (*n* = 60), and internal test (*n* = 57) subsets. The validation set was intended for checkpoint and hyperparameter selection; the held-out ADNI test set and external cohorts were intended for final evaluation.

**Figure 3:**
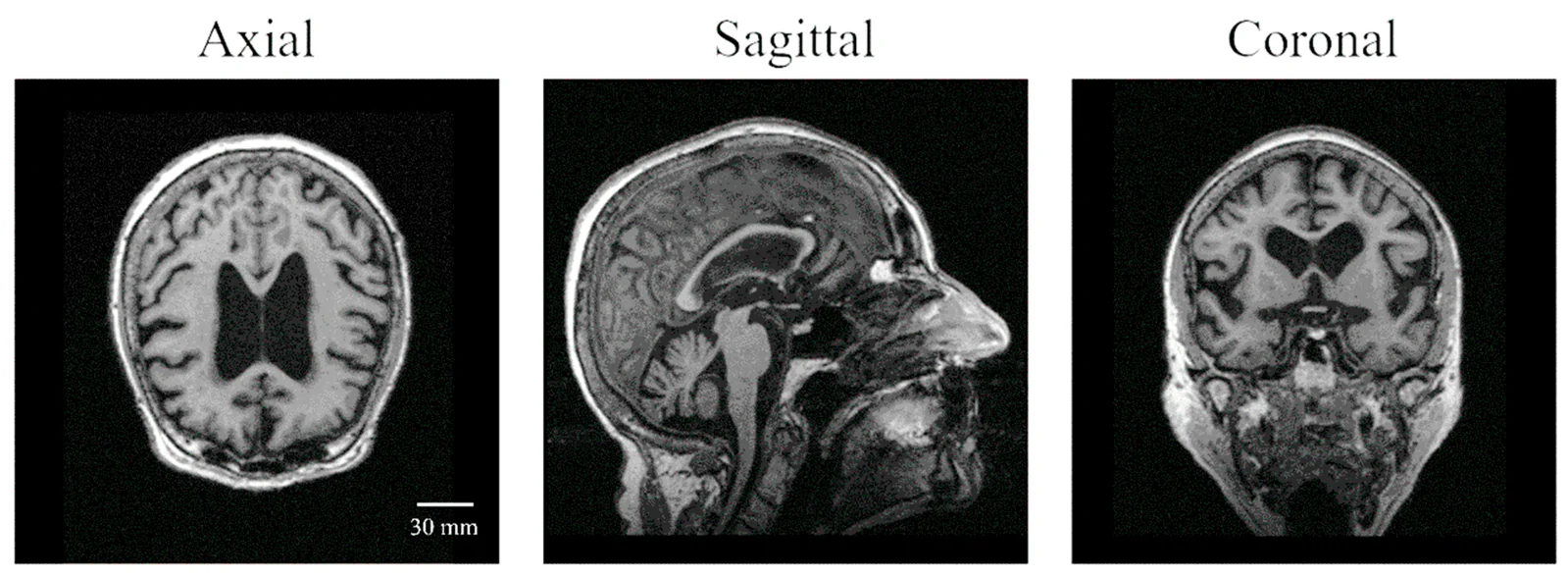
Representative axial, sagittal, and coronal T1-weighted images from ADNI.

We used the clinically defined diagnostic labels from each parent cohort without re-adjudication. ADNI applied NIA-AA criteria; AIBL used multidisciplinary consensus; FHS followed DSM-IV and NINCDS-ADRDA; and NACC employed standardized UDS consensus diagnoses. Mixed and non-AD dementias were excluded where specified by the parent-cohort definitions. Amyloid PET and CSF biomarker data were available in subsets of ADNI and AIBL but were not used for sample selection or model development. Participants with missing MMSE scores were excluded. Because stage-wise exclusion counts were not systematically retained in the original retrospective preprocessing logs, a fully reconstructed exclusion flow cannot be reported without risking inaccurate counts; we therefore report the final analyzable cohorts and all available eligibility criteria transparently, and Figure 3 shows representative T1-weighted MRI images from the ADNI cohort.

### 3.2. Primary Evaluation and Metrics

Performance was recorded after each of five refinement iterations. The primary analysis used the model selected exclusively from ADNI validation data: iteration 3, the checkpoint with the lowest validation loss, and a classification threshold of 0.5. These choices were locked before final interpretation and applied unchanged to the held-out ADNI test set and all external cohorts. The remaining iterations are presented as sensitivity analyses to characterize the effect of refinement stage and were not used for cohort-specific model selection.

We evaluated sensitivity, specificity, F1 score, Matthews correlation coefficient (MCC), receiver operating characteristic area under the curve (ROC AUC), and precision–recall area under the curve (PR AUC). Threshold-dependent metrics used the locked threshold of 0.5. Given the differing AD prevalence across cohorts, F1 scores are interpreted together with sensitivity, specificity, and MCC rather than as a stand-alone measure.

### 3.3. Classification Performance

Table 3 summarizes classification performance across all five refinement iterations, with iteration 3 designated as the validation-selected primary model. On the held-out ADNI test set, iteration 3 achieved sensitivity 0.889, specificity 0.841, F1 score 0.853, and MCC 0.726. Without retraining or threshold adjustment, the same model achieved sensitivity/specificity/F1/MCC of 0.871/0.831/0.807/0.685 in NACC, 0.897/0.822/0.765/0.667 in FHS, and 0.855/0.891/0.707/0.653 in AIBL. These results demonstrate that the validation-selected weakly supervised model retained balanced performance across three independent external cohorts.

**Figure 4:**
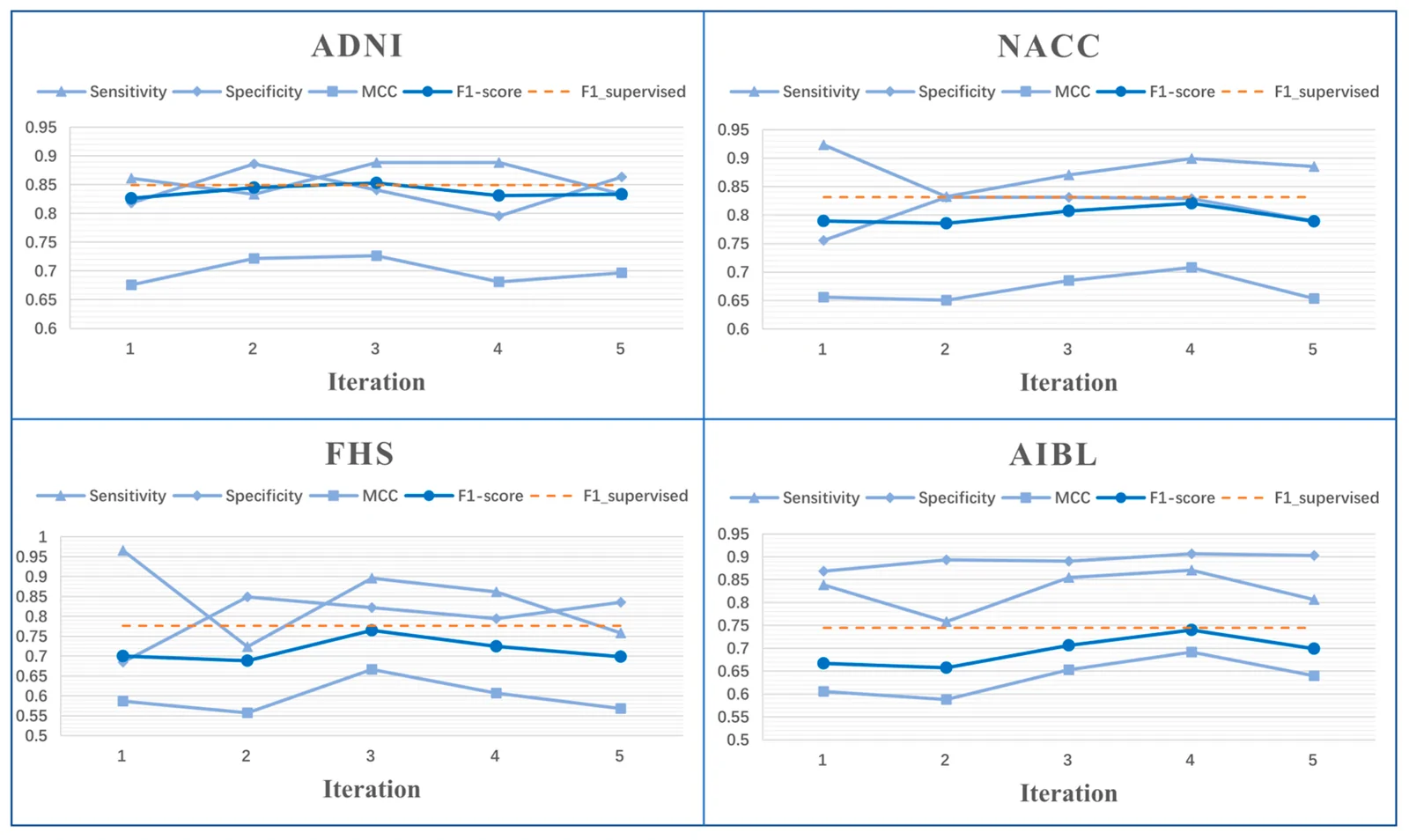
Sensitivity, specificity, F1 score, and MCC trajectories across five refinement iterations. Iteration 3 is the validation-selected primary model; the remaining iterations are shown as sensitivity analyses. The dashed supervised reference from the original implementation is retained only as descriptive context and is not used for model selection or formal inferential comparison. MCC, Matthews correlation coefficient; ADNI: Alzheimer’s Disease Neuroimaging Initiative; NACC: National Alzheimer’s Coordinating Center; FHS: Framingham Heart Study; AIBL, Australian Imaging, Biomarkers and Lifestyle Flagship Study of Ageing.

Figure 4 plots the trajectories of sensitivity, specificity, F1 score, and MCC across the five refinement iterations. Performance was broadly stable across the refinement trajectory, with ADNI F1 scores ranging from 0.827 to 0.853 and external-cohort F1 scores ranging from 0.657 to 0.821. The validation-selected iteration 3 provided a favorable balance of sensitivity and specificity across cohorts and was therefore used for the primary interpretation. Because F1 is prevalence-dependent and cohort composition differed substantially, cross-cohort comparisons are interpreted in conjunction with sensitivity, specificity, and MCC rather than as direct measures of relative cohort difficulty.

Importantly, no cohort-specific iteration was selected. Iteration 3 and the 0.5 threshold were fixed from ADNI validation data and then transferred unchanged to the ADNI test, NACC, FHS, and AIBL. This locked evaluation strategy avoids optimizing the model separately on any held-out or external cohort and strengthens the interpretation of the multi-cohort results.

**Figure 5:**
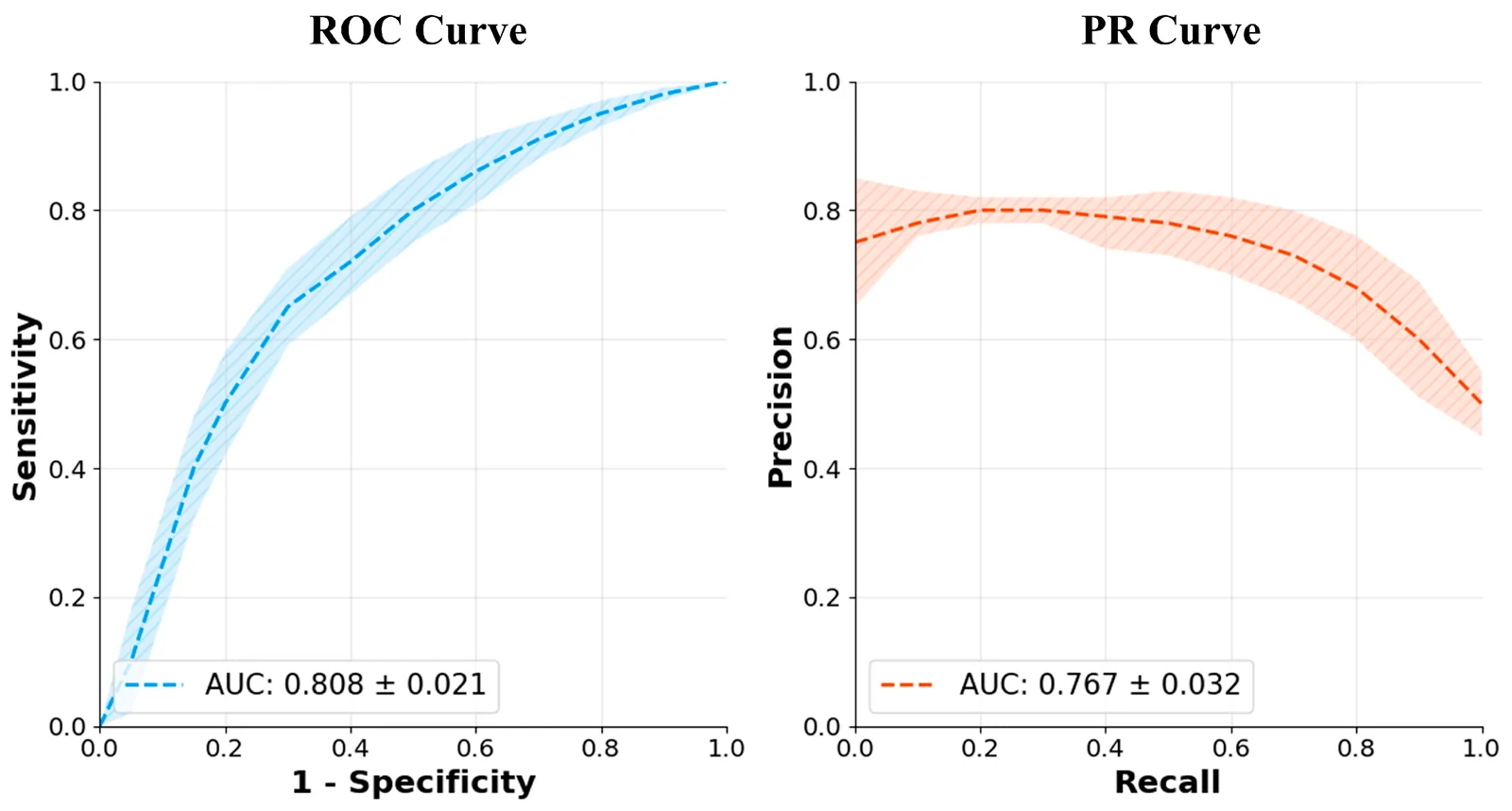
Receiver operating characteristic (ROC) and precision–recall (PR) curves for the ADNI test set. ROC AUC = 0.808 and PR AUC = 0.767. Shaded bands and the ± values displayed within the original panels represent bootstrap-derived variability; they are not interpreted here as formal 95% confidence intervals because archived lower and upper limits were unavailable.

For the ADNI test set, the reported receiver-operating-characteristic and precision–recall analyses yielded ROC AUC = 0.808 and PR AUC = 0.767 (Figure 5). The uncertainty displayed in the original figure was generated by bootstrap resampling and is retained as graphical variability. Because the archived output does not contain the exact lower and upper interval limits, we do not relabel these values as formal 95% confidence intervals. The AUC results complement the locked-threshold metrics and support useful discrimination on the internal test set.

The FCN architecture was adapted from Qiu et al. [15] and trained from random initialization. Accordingly, the contribution of the present work lies in the weak-supervision schedule rather than transfer of pretrained weights or introduction of a new backbone. The results show that clinically generated pseudo-labels and diagnosis-guided refinement can be integrated with an established FCN–MLP architecture and transferred to independent cohorts without retraining.

Overall, the validation-selected weakly supervised FCN–MLP achieved consistent separation of cohort-defined AD and NC groups in the held-out ADNI test set and three external cohorts without external retraining or cohort-specific threshold adjustment. These findings support the feasibility of transferring clinically derived supervisory information into an MRI-only inference model across heterogeneous datasets.

## 4. Discussion

This study demonstrates the feasibility of a weakly supervised MRI-classification strategy in which clinically generated pseudo-labels initialize an imaging model and partially weighted diagnostic information subsequently anchors iterative self-training. A recent *Nature* review characterizes weak supervision as learning from imperfect, partial, or noisy supervisory signals [6]; under this broad and widely used definition, the present framework is appropriately described as weakly supervised while remaining explicit that ground-truth labels contribute 20% to each refinement target. The validation-selected model was applied unchanged to the ADNI test, AIBL, FHS, and NACC, providing a consistent basis for multi-cohort evaluation.

The imaging backbone—a patch-based FCN with an MLP on selected voxel probabilities—was adapted from Qiu et al. [15], including 47 × 47 × 47 patches, whole-brain probability maps, and a spatial MLP. The methodological contribution is the supervision strategy: clinical variables are converted into pseudo-labels that initialize MRI learning, model-generated probabilities remain the dominant component of subsequent targets, and a 20% diagnostic anchor constrains iterative refinement. This approach does not seek to eliminate diagnostic labels; instead, it restructures how direct and indirect supervision are combined during optimization. At inference, final classification is based on MRI-derived features rather than clinical variables.

Several aspects of the findings are noteworthy. First, MMSE is associated with clinical diagnosis and therefore provides an informative, though not independent, supervisory signal. The purpose of the clinical teacher is precisely to convert this routinely available information into pseudo-labels that can guide MRI representation learning. Second, the use of an 80% model-prediction component during refinement preserves a substantial self-training signal while the 20% diagnostic component reduces the risk of uncontrolled label drift. Third, despite marked differences in cohort composition and prevalence, the locked iteration-3 model achieved F1 scores of 0.807 in NACC, 0.765 in FHS, and 0.707 in AIBL without retraining. This cross-cohort transfer is a practical strength of the framework, although it should not be interpreted as evidence of universal clinical generalizability.

This study also has limitations. The task was restricted to binary AD-versus-NC classification and did not include MCI, atypical presentations, or differential diagnosis. AIBL and FHS contained relatively few AD cases, and prevalence differed across cohorts. Complete lower/upper bootstrap interval limits, formal calibration and decision-curve analyses, and detailed error stratification were not available in the archived analysis. The original preprocessing logs also did not retain exact counts for missing-MMSE exclusions or manually corrected registrations. These limitations affect the precision and clinical scope of the conclusions but do not alter the central proof-of-concept finding that a clinically initialized weak-supervision strategy can support MRI classification across multiple independent cohorts.

Future work can extend this proof of concept through matched supervised and clinical-only baselines, formal calibration and decision-curve analysis, detailed error and subgroup analyses, site-aware validation, and prospective evaluation in MCI and diagnostically heterogeneous populations. Such studies would clarify the incremental contribution of each supervisory component and the potential role of the framework in clinically more challenging settings.

## 5. Conclusions

This retrospective proof-of-concept supports the feasibility of weakly supervised MRI-based classification of cohort-defined AD and NC groups using clinical pseudo-labels. The framework combines imperfect clinical pseudo-label initialization with iterative self-training in which model-generated probabilities contribute 80% and diagnostic labels contribute a 20% anchor. Using ADNI validation data only, iteration 3 and a threshold of 0.5 were selected and then applied unchanged to the held-out ADNI test set and three external cohorts, yielding F1 scores of 0.853, 0.707, 0.765, and 0.807 in ADNI, AIBL, FHS, and NACC, respectively. These findings show that clinically derived supervisory information can be transferred into an MRI-based classifier that maintains useful performance across heterogeneous cohorts without external retraining. The study therefore provides a transparent weak-supervision framework and a practical foundation for future evaluation in earlier and more diagnostically complex disease stages.

## Figures and Tables

**Table 1:** Auditable data lineage for each model component and evaluation stage.

Stage	Participants/Data	Inputs	Diagnostic-Label Use	Implementation Role
Clinical MLP training	40 ADNI training participants (20 AD, 20 NC)	Age, sex, MMSE	True AD/NC labels used as targets	Initial clinical teacher trained on a balanced 40-participant subset.
Initial target generation	Remaining 260 ADNI training participants	Age, sex, MMSE	Diagnosis not used to generate the initial prediction	Clinical MLP generates the initial pseudo-label probability/class.
Initial FCN training	MRI from the same 260 participants	47 × 47 × 47 MRI patches	Clinically generated initial targets used	Participant-level clinical pseudo-labels are inherited by sampled MRI patches.
Iterative refinement	Same 260 ADNI training participants	Previous MRI probability and true diagnosis	True label contributes 20% at every update	P(t) = 0.8p(t − 1) + 0.2y; the fused probability is binarized at 0.5 and used as a hard cross-entropy target for the next refinement round.
Validation	60 ADNI validation participants	MRI-model outputs	Labels used for validation	Iteration 3 selected by validation F1; checkpoint retained by validation loss; threshold 0.5 selected on validation data.
Internal test	57 ADNI test participants	Locked model outputs	Labels used only for final performance evaluation	Locked iteration-3 model and threshold applied without test-driven model selection.
External evaluation	AIBL *n* = 182; FHS *n* = 102; NACC *n* = 265	Locked model outputs	External labels used only for evaluation	Locked iteration-3 model and threshold applied unchanged to all external cohorts.

**Table 2:** Study cohorts and participant characteristics.

Dataset	Group	Age, Median [Range]	Sex, Male, *n* (%)	MMSE, Median [Range]
ADNI	NC (*n* = 229)	76 [60, 90]	119 (51.96)	29 [25, 30]
	AD (*n* = 188)	76 [55, 91]	101 (53.72)	23.5 [18, 28]
AIBL	NC (*n* = 152)	72 [60, 92]	68 (44.74)	29 [25, 30]
	AD (*n* = 30)	73 [55, 93]	12 (40.00)	21 [6, 28]
FHS	NC (*n* = 73)	73 [57, 100]	37 (50.68)	29 [22, 30]
	AD (*n* = 29)	81 [67, 94]	12 (41.38)	25 [10, 29]
NACC	NC (*n* = 167)	74 [56, 94]	59 (35.33)	29 [20, 30]
	AD (*n* = 98)	77 [55, 95]	45 (45.92)	22 [0, 30]

**Table 3:** Classification performance across five diagnosis-guided refinement iterations for ADNI, NACC, FHS, and AIBL. Iteration 3 was selected using ADNI validation data and constitutes the primary model; it was applied unchanged to all test cohorts. The other iterations are shown as sensitivity analyses.

	**Dataset**	**ADNI**				**NACC**			
**Iteration**		Sensitivity	Specificity	F1 score	MCC	Sensitivity	Specificity	F1 score	MCC
1		0.861	0.818	0.827	0.676	0.923	0.756	0.789	0.656
2		0.833	0.886	0.845	0.722	0.833	0.831	0.786	0.651
3		0.889	0.841	0.853	0.726	0.871	0.831	0.807	0.685
4		0.889	0.795	0.831	0.681	0.9	0.829	0.821	0.708
5		0.833	0.864	0.833	0.697	0.885	0.789	0.789	0.653

	**Dataset**	**FHS**				**AIBL**			
**Iteration**		Sensitivity	Specificity	F1 score	MCC	Sensitivity	Specificity	F1 score	MCC
1		0.966	0.685	0.7	0.587	0.839	0.869	0.667	0.606
2		0.724	0.849	0.689	0.557	0.758	0.894	0.657	0.588
3		0.897	0.822	0.765	0.667	0.855	0.891	0.707	0.653
4		0.862	0.795	0.725	0.607	0.871	0.906	0.74	0.692
5		0.759	0.836	0.698	0.569	0.806	0.903	0.699	0.64

## Data Availability

The data analyzed in this study are available from the respective ADNI, AIBL, FHS, and NACC repositories, subject to application, approval, and the relevant data-use agreements. The sample data used for the experiments in this study are openly available on GitHub at https://github.com/xiaoronghust/AD (accessed on 5 July 2026).

## Abbreviations

The following abbreviations are used in this manuscript:

AD	Alzheimer’s disease
CSF	cerebrospinal fluid
PET	positron emission tomography
MRI	magnetic resonance imaging
WSL	weakly supervised learning
ADNI	Alzheimer’s Disease Neuroimaging Initiative
MMSE	Mini-Mental State Examination
AIBL	Australian Imaging, Biomarkers and Lifestyle Flagship Study of Ageing
FHS	Framingham Heart Study
NACC	National Alzheimer’s Coordinating Center
NC	normal cognition
GT	ground truth
FCN	fully convolutional network
MLP	Multilayer Perceptron
AUC	area under the curve
MCC	Matthews correlation coefficient
